# Reconstruction of an Anterior Cervical Necrotizing Fasciitis Defect Using a Biodegradable Polyurethane Dermal Substitute

**Published:** 2017-01-25

**Authors:** Marcus JD Wagstaff, Yugesh Caplash, John E Greenwood

**Affiliations:** ^a^Adult Burn Centre, Royal Adelaide Hospital, Adelaide, South Australia, Australia; ^b^Department of Plastic and Reconstructive Surgery, Royal Adelaide Hospital, Adelaide, South Australia, Australia

**Keywords:** BTM, necrotizing fasciitis, cervical reconstruction, dermal matrix, biodegradable polyurethane

## Abstract

**Introduction:** Although we have previously described the use of a novel polyurethane biodegradable dermal substitute in the reconstruction of 20 free flap donor sites, and extensive cutaneous defects, including a large area of exposed calvarium secondary to burn injury, our experience with this material now extends to 35 free flap donor site reconstructions and 13 major or complex burns. **Methods:** The polyurethane material (NovoSorb BTM; PolyNovo Biomaterials Pty Ltd, Port Melbourne, Victoria, Australia) was recently employed in another complex wound scenario, implanted into a large anterior cervical cutaneous and soft-tissue defect remaining after serial radical debridement for necrotizing fasciitis. **Results:** Implantation, integration, delamination, and split-skin graft application proceeded without complication, mirroring our previous experience in other wounds (including major burns). The result was a robust, supple, mobile, and well-contoured reconstruction over the deep tissues of the neck. The functional and cosmetic outcomes exceeded all expectation. **Discussion:** The wound environment created after necrotizing fasciitis infection and debridement is austere. In this particular case, reconstructive options were limited to large free flap repair, skin graft alone, and skin graft augmented by commercially available collagen/glycosaminoglycan dermal matrix. Each option was discarded for various reasons. Our previous success with NovoSorb BTM, developed at our center, prompted its use following regulatory approval. The patient was physiologically stronger after the temporization afforded by the biodegradable temporizing matrix over 4 weeks of integration. **Conclusion:** This is the first description of the successful use of an entirely synthetic biodegradable dermal substitute for the reconstruction of both necrotizing fasciitis and an anterior cervical defect.

There are several published series and case reports on the successful use of the biological dermal regeneration template Integra (Integra LifeSciences Corp, Plainsboro, NJ) in the reconstruction of trunk and limb wounds postdebridement of necrotizing fasciitis or meningococcal septicemia.^1^^-^7 Integra and similar dermal substitutes, such as Matriderm (Dr Suwelack Skin and Health Care AG, Billerbeck, Germany), have also been described in the elective reconstruction of defects after postburn neck contracture release.^8^^-^10

The choice of cervical skin and soft-tissue reconstruction following contracture release is influenced by the extent of the release and defect produced, as well as available technical and financial resources. Commonly, these are smaller defects from skin and scar excision alone, which do not expose the deep structures of the neck and may be reconstructed with a full-thickness skin graft. In larger areas without deep vessels exposed, a split-thickness skin graft may suffice; otherwise, pre-expanded regional and free flap reconstructions are also recognized standards of care.^11^^,^^12^ In extensive defects, robust cover of exposed deep structures with effective contouring of the submental and cervical subunits has been achieved with thinned fasciocutaneous free flaps such as anterolateral thigh or thoracodorsal artery perforator flaps.^13^ However, such techniques are complex, require microvascular surgical expertise, and leave a donor site usually requiring secondary split-thickness skin graft reconstruction. Interestingly, there is no description of the use of a dermal substitute for reconstruction of a cervical soft-tissue defect following necrotizing fasciitis. This case combines the reconstructive challenges of an extensive skin and soft-tissue defect created after radical surgical debridement, and anterior cervical soft-tissue reconstruction, addressing the need for robust cover of the deep structures with a technically simpler procedure than a free flap.

We have developed a bilayer biodegradable polyurethane dermal substitute (biodegradable temporizing matrix [BTM]) and trialed its use in debrided deep dermal and full-thickness 20% to 50% total body surface areas burn wounds (unpublished data) and as part of this development we have applied it to iatrogenic surgical wounds.^14^^,^^15^ We have also reported its use in coverage of an extensive exposed calvarial defect following full-thickness burn excision.^16^ Its use in this case was intended to offer a robust cover of the deep structures of the neck with reduced post–skin graft scar contracture, while due to its synthetic composition, remaining inert in the presence of infection.

## CASE PRESENTATION

**Figure 1:**
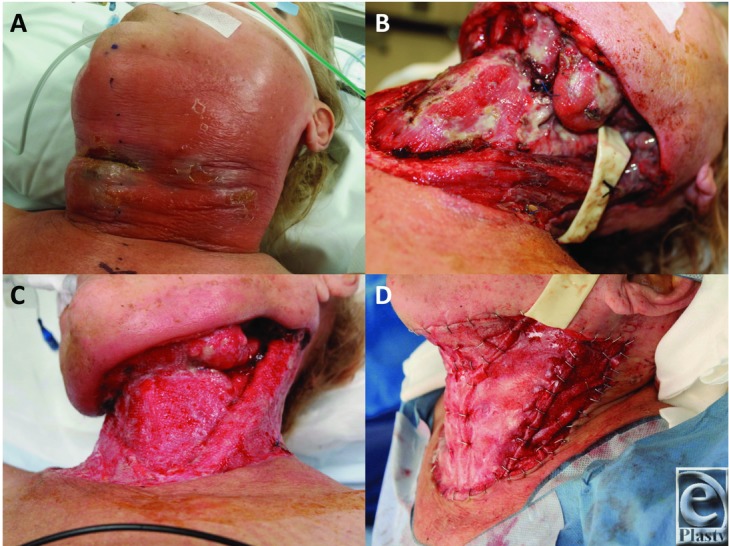
(a) Initial presentation. Gross swelling of the left face and neck tissues with inflammation and epidermolysis indicative of deep infection. (b) Following the initial debridement on the day of presentation. A drain has been left in the left parotid region (the origin of the infection). Debridement has exposed the strap muscles, sternocleidomastoids, and the left submandibular gland. (c). Eleven days later, the infection has been eradicated following repeated debridement and light granulations can be seen. (d) The granulations were gently dermabraded and the biodegradable temporizing matrix was applied with staples.

A 56-year-old woman presented to her local hospital with a 1-day history of pain and swelling to the soft tissues to the anterior neck. This was accompanied by symptoms of fever and malaise. A severe, acute, soft-tissue infection with a threatened airway was diagnosed and she was intubated and transferred to our hospital (Fig 1a). On arrival, she was in established septic shock and requiring inotrope support to maintain her hemodynamic stability. She was transferred immediately to the operating theater where she underwent radical debridement of necrotizing fasciitis of the skin and soft tissues of the neck. Pus was found in the left parapharyngeal space tracking around the mandible, over the masseter, tracking between the middle and superficial layers of the deep cervical fascia. A large amount of pus was present between the left parotid gland and the masseter muscle. Necrotic fat was widespread. The necrotic skin, platysma, fascia and fat were debrided, exposing the deep structures of the neck (Fig 1b). All tissues were irrigated. Both external carotid arteries were ligated. She was administered intravenous clindamycin, meropenem, and vancomycin according to our necrotizing fasciitis protocol. Pus and deep fascia subsequently cultured a mixed growth of microaerophilic streptococci, mixed anaerobes, and *Candida albicans*. Antibiotic regimens were altered according to sensitivities.

Twenty-four hours later, more necrotic skin and tissue over the sternocleidomastoid and strap muscles were debrided and further periparotid pus and pus extending to the chest was washed out in theater. The third and fourth look operations on days 3 and 4, respectively, revealed further pus draining from the left parotid gland, which was irrigated. Inotropes were ceased on day 4 as she became more hemodynamically stable. Further wound checks in theater were performed on days 6 and 7. At this time, we began to consider options for reconstruction. The wound dimensions at this time were 42 × 16 cm, reaching to level V bilaterally and exposing sternocleidomastoids, carotid sheaths, and the left submandibular gland (Fig 1c). We considered a free anterolateral thigh flap; however, a very large flap would be required and the usually favored recipient vessels bilaterally were in the zone of injury, which would place the microsurgical anastomoses at risk. She had also developed a concomitant ventilator-acquired pneumonia, culture-positive for *Stenotrophomonas maltophilia*, and we were concerned about a long anesthetic regimen and how this might cause further deterioration. We considered reconstructing the area with the dermal substitute Integra; however, in a previously infected wound around the neck, we were concerned that the biological components of Integra might predispose it to become infected close to the common/internal carotid vessels, posing a significant risk of further systemic upset and carotid blowout to the patient.

We applied to the Australian regulatory body, the Therapeutic Goods Administration (TGA), for permission to use the NovoSorb BTM (PolyNovo Biomaterials Pty Ltd, Port Melbourne, Victoria, Australia), a 2-mm thick, totally synthetic polyurethane dermal substitute foam with an overlying nonbiodegradable removable seal. We have previously used the BTM successfully in clinical pilot trials for moderate/severe burn wounds (unpublished) and free flap donor sites,^14^^,^^15^ and subsequently under the TGA's Authorised Prescriber Scheme. We applied for permission to use the BTM as a single case under another regulatory permit, the Special Access Scheme, which was granted.

On day 11 postadmission, she attended the operating theater for BTM application. The wound edges were refreshed by sharp excision. The wound bed was irrigated and cleaned and the BTM cut to fit into 4 sections (1 piece for each level V, 1 for the anterior neck, and 1 for the submental unit) and applied with staples (Fig 1d). Quilting staples were applied anteriorly. The BTM was dressed with Mepitel (Mölnlycke Health Care, Hamburg, Germany), overlaid with Acticoat (Smith & Nephew, Hull, UK), secured in position with Hypafix (BSN Medical, Mount Waverley, Victoria, Australia).

**Figure 2:**
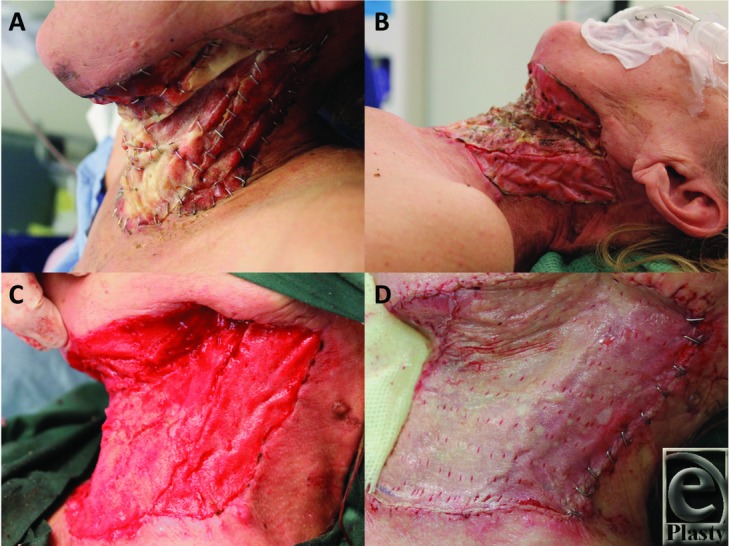
(a) After 10 days, BTM integration proceeds with many areas adopting a pink color. (b) By 34 days postapplication, the BTM is fully integrated. (c) The nonbiodegradable seal is removed (delamination) to reveal a vascularized neodermis within the polyurethane foam. (d) Following gentle dermabrasion to refresh the surface, a fenestrated sheet autograft was applied. BTM indicates biodegradable temporizing matrix.

**Figure 3:**
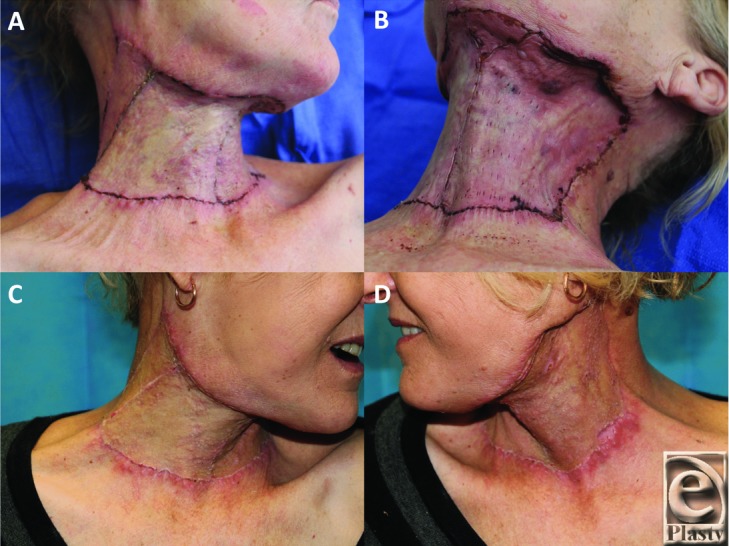
(a,b) The graft at 4 days was robust and massage and moisturization commenced. (c,d) At day 14 postgrafting, range of movement was excellent.

Dressing changes were performed twice weekly on the ward, replacing the Mepitel and Acticoat on each occasion. A hard (Philadelphia) collar was fitted to prevent early neck contracture, which the patient was compliant with. At the first 2 dressing changes, any nonadhered BTM was secured into position with staples and redundant BTM was trimmed with scissors. The BTM continued to adhere and integrate without incident. At 2 weeks postimplantation, when the BTM was fully adherent and Mepitel was omitted from the dressing regimen, leaving only Acticoat and Hypafix to be replaced. By day 34 postimplantation, the BTM was salmon pink in color (Figs 2a and 2b), with a capillary refill demonstrable through the seal on digital pressure. Under general anesthetic, the BTM was delaminated, the surface lightly refreshed by dermabrasion (Fig 2c), and a 0.010-in split-skin graft (SSG) was harvested from the thigh, hand fenestrated, and applied to the integrated defect using steel skin staples (Fig 2d). The SSG was dressed with Jelonet (Smith & Nephew, Hull, UK), betadine-soaked cotton gauze, and Hypafix. The first dressing change was performed at the bedside on day 4 post–skin graft application (Figs 3a and 3b). The graft had taken well, with 2 small (<1-cm diameter) underlying hematomas that were evacuated by small incisions in the graft, with no subsequent graft loss. These small areas were redressed, and the rest of the SSG was robust to shear and ready for moisturizing. She was discharged for rehabilitation 2 days later. She suffered no secondary wound breakdowns, and the graft has continued to mature and permit a full range of neck movement (Figs 3c and 3d). Videos 1 and 2 show the range of motion and condition of the scarring 3 months postimplantation.

**Video 1:** At day 86 post–graft application, an excellent range of motion is observed in all directions.

**Video 2:** At the same day 86 postgrafting time point, the new “skin” is soft, supple, and mobile.

## DISCUSSION

This is the first use of the BTM in a surgical wound outside of the TGA authorized indications of free flap donor site repair and burn wound closure. We considered several factors in choosing a reconstruction for an anterior cervical skin and soft-tissue defect of this size. Although the sternocleidomastoid muscles were left intact and would successfully take a skin graft, the deep structures of the neck (internal jugular vein, submandibular glands, common carotid arteries, etc) require more robust cover. A large free flap would afford this, and we would otherwise have elected to use an anterolateral thigh flap. This, however, would commit the patient to a significant defect on her thigh, requiring split-thickness skin graft reconstruction. In addition, the internal jugular veins and carotid vessels bilaterally were involved in the zone of injury, which could compromise the reliability of any microsurgical anastomosis. At the time when the neck wound was clean enough to consider reconstruction, the patient was still resident on the intensive care unit, and although she was hemodynamically stable, she was suffering from a concomitant chest infection. A long anesthetic regimen, which a free flap procedure would have required and put her under significant risk of further deterioration. At this time, the wound had started to granulate and there was already some evidence of wound contraction, which if allowed to progress unchecked would cause considerable fixed flexion and restriction of neck mobility. The wound was too large in dimension for any single regional pedicled flap reconstruction, and a multiple flap procedure, such as bilateral pectoralis musculocutaneous flaps, would also have left her with considerable donor site deformities.

The BTM has been shown to integrate and take an SSG with favorable scarring results on objective measurement. Given our developmental experience with the BTM as authorized prescribers of an unapproved product in the reconstruction of free flap donor sites and extensive burn wounds, we extrapolated its use to this indication.

The BTM integrated without complication over 4 weeks. At 4 weeks, the previously visible foam architecture had become obliterated with salmon pink/orange tissue, which exhibited blanching on digital pressure and capillary refill on release. We then scheduled the delamination and split-skin grafting procedure the following week at day 34 postimplantation. Delamination revealed a uniformly red, integrated matrix, which bled after light dermabrasion. Three small areas of nonintegration (each <5-mm diameter) revealed the thickness of the remaining integrated BTM.

The hand-fenestrated split-thickness skin graft took to the wound bed without complication and was robust to massaging with moisturizer at day 4 postapplication after the first dressing change. This degree of adherence at 4 days is subjectively considerably greater than we experience with a similar skin graft placed directly on the wound bed.

Given some degree of wound contraction and graft hypertrophy is inevitable over the first 3 months in this functionally and aesthetically sensitive area, she was referred to our occupational therapist for scar therapy with compression garments and silicone treatment. She has also been instructed in vigorous scar massage with moisturizer and stretching exercises to maintain range of movement. Her SSG donor site healed without complication.

## CONCLUSION

The results of this case support the use of the BTM in nonelective complex wounds. The BTM affords a totally synthetic dermal replacement strategy for the deep structures of the neck. Its use may therefore also be extrapolated to surgical neck scar contracture release, postdebridement of trunk and limb necrotizing fasciitis, and other deep and/or complex posttraumatic wounds that would benefit functionally and aesthetically from thicker, more robust cover than SSG alone can achieve.
